# Geriatric Patients Undergoing Outpatient Surgery in the United States: A Retrospective Cohort Analysis on the Rates of Hospital Admission and Complications

**DOI:** 10.7759/cureus.20607

**Published:** 2021-12-22

**Authors:** Rahul Chaturvedi, Kruti Patel, Brittany N Burton, Rodney A Gabriel

**Affiliations:** 1 Anesthesiology, University of California San Diego School of Medicine, La Jolla, USA; 2 Anesthesiology and Perioperative Medicine, University of California Los Angeles David Geffen School of Medicine, Los Angeles, USA

**Keywords:** general and laparoscopic surgery, hospital admission, ambulatory surgery, post-operative morbidity, geriatrics and internal medicine

## Abstract

Introduction: This study is a retrospective cohort analysis that describes key clinical outcomes in elderly individuals who undergo outpatient surgical procedures. In particular, we report same-day admission, 30-day mortality, 30-day complications, and 30-day readmission rates for three separate age groups undergoing frequent outpatient, general surgical procedures.

Methods: Patients ≥18 years old who underwent the 10 most common outpatient surgical procedures in the National Surgical Quality Improvement Program database from 2017 to 2019 and who underwent general anesthesia were included in the study. The primary outcome of interest was hospital admission, defined as hospital length of stay >0 days. Secondary outcomes of interest included 30-day readmission, 30-day mortality, and 30-day postoperative complications. The primary exposure variable of interest was age, which was divided into <65 years of age (reference cohort), 65-79 years of age, and ≥80 years of age. For univariate analysis, to measure differences in the outcomes and patient characteristics, we used chi-squared tests. Our primary method of analysis was multivariable logistic regression.

Results: Those who were ≥80 and 65-79 years of age compared to <65 years of age had higher odds of same-day admission, 30-day mortality, composite postoperative complications, and readmission. Patients who were ≥80 years old had higher odds of same-day admission for laparoscopic cholecystectomy, partial mastectomy, laparoscopic inguinal hernia repair, inguinal hernia repair, umbilical hernia repair, laparoscopic removal of adnexal structures, and lumbar laminotomy.

Conclusion: Increasing age, particularly greater than 80 years or older and 65-79 years of age group, is associated with an increased rate of same-day hospital admissions and complications after ambulatory surgery.

## Introduction

In 2018, there were 52 million people aged 65 years and older according to the Census Bureau’s Vintage Population Estimates. Their share of the population grew from 12.4% in 2000 to 16.0% in 2018 [1]. By the year 2034, 77 million citizens of the United States will be above the age of 65, surpassing the 76.5 million who are under 18 years [2]. Given this rise in the aging population, physicians and healthcare systems are caring for a larger proportion of elderly patients.

Ambulatory surgery has been increasing since the 1980s. According to the Centers for Disease Control and Prevention National Health Statistics report, there has been approximately a 300% increase in the rate of visits to freestanding ambulatory surgery centers [3]. Of the 70+ million procedures performed annually here in the United States, more than 30% occur at freestanding ambulatory surgical centers, where no hospital or emergency department is attached and patients are expected to go home the same day (or within 24 hours) [4]. The number of ambulatory surgeries in the aging population has thereby increased as well.

Although it has been previously documented that mortality and complications are greater with major surgeries in older populations, less is known about the association of elderly age on outcomes following ambulatory surgical procedures [5]. The purpose of this study is to perform a retrospective cohort analysis to describe clinical outcomes in elderly individuals who undergo outpatient surgical procedures. In particular, we report same-day admission, 30-day mortality, 30-day complications, and 30-day readmission rates for three separate age groups undergoing frequent outpatient, general surgical procedures. We hypothesized that those aged ≥80 years old would not have differences in postoperative outcomes for outpatient surgical procedures than the <65-year-old group, given the nature of the surgeries being performed.

## Materials and methods

In this retrospective study, patient data were de-identified and thus ethical approval was not required by our institutional review board, Human Research Protections Program. The American College of Surgeons (ACS) National Surgical Quality Improvement Program (NSQIP) registry was used to extract all patient records. To prevent bias in sampling, the ACS NSQIP database undergoes a systemic sampling process called the eight-day cycle, giving each case an equal chance of being selected from each day of the week. This manuscript adheres to the Enhancing the Quality and Transparency of Health Research guidelines.

The population of interest was defined as patients ≥18 years old who underwent the top 10 most common outpatient surgical procedures in the ACS NSQIP database (shoulder arthroscopy, knee arthroscopy, lumbar laminotomy, laparoscopic appendectomy, laparoscopic cholecystectomy, laparoscopic inguinal hernia repair, laparoscopic removal of adnexal structures, partial mastectomy, inguinal hernia repair, and umbilical hernia repair) from 2017 to 2019 and who received general anesthesia. The cases were identified with Current Procedural Terminology (CPT) codes of 29827 (shoulder arthroscopy), 29881 (knee arthroscopy), 63030 (lumbar laminotomy), 44970 (laparoscopic appendectomy), 47562 (laparoscopic cholecystectomy), 49650 (laparoscopic inguinal hernia repair), 58661 (laparoscopic removal of adnexal structures), 19301 (partial mastectomy), 49505 (inguinal hernia repair), and 49585 (umbilical hernia repair). Inclusion criteria included a case that was designated as outpatient, aged ≥18 years old, was one of the 10 described surgical procedures, and general anesthesia was the primary anesthetic.

The primary outcome of interest was hospital admission, defined as hospital length of stay >0 days. Secondary outcomes of interest included 30-day readmission, 30-day mortality, and 30-day postoperative complications. Thirty-day postoperative complications were defined as the composite occurrence of superficial surgical site infection, wound disruption, pneumonia, reintubation, pulmonary embolism, urinary tract infection, stroke, myocardial infarction, or deep vein thrombosis. The primary exposure variable of interest was age, which was divided into those <65 years of age (reference cohort), 65-79 years of age, and ≥80 years of age. Additional covariates collected were sex, body mass index (divided by ≥40kg/m^2^ or less), diabetes mellitus, active smoking history, preoperative dyspnea, functional status (classified as independent or dependent in activities of daily living), severe chronic obstructive pulmonary disease, hypertension, preoperative steroid use, and preoperative bleeding disorder. We removed all cases with missing data for the following variables: sex, age, body mass index, and functional status. All other covariates did not contain missing data.

Statistical analysis

Statistical analysis was performed using R, a software environment for statistical computing (R version 3.6.1, R Foundation for Statistical Computing, Vienna, Austria). For univariate analysis, to measure differences in the outcomes and patient characteristics, we used chi-squared tests. Our primary method of analysis was multivariable logistic regression. The dependent variable was same-day hospital admission. The main independent variable of interest was the age cohort (described above). We controlled for other confounders including sex, body mass index, diabetes, active smoking history, preoperative dyspnea, functional status, chronic obstructive pulmonary disease, hypertension, steroid use, and bleeding disorders. All covariates were included in the regression. We report the odds ratio (OR) and its 95% confidence interval (CI). We performed a similar analysis for our secondary outcomes, in which the dependent variables were either 30-day readmission, 30-day mortality, or 30-day postoperative complications. We considered a p-value < 0.017 to be statistically significant to control for the number of age groups we studied.

## Results

Data extraction from the ACS NSQIP database yielded 1,368,738 adult patients who underwent outpatient surgery from 2017 to 2019, of whom 1,248,057 received general anesthesia for those procedures. Of those adults who received general anesthesia for the above surgical procedures, 395,145 underwent one of the 10 most frequently performed outpatient surgical procedures outlined above. After removing cases with missing data, we included 383,468 patients in the final analysis.

Of these, 295,971 (77.2%) were <65 years old, 75,629 (19.7%) were between 65 and 79 years old, and 11,868 (3.1%) were ≥80 years old. Among patients who were ≥80 years old, 2,408 (20.3%) had a same-day admission, 29 (0.2%) experienced 30-day mortality, 258 (2.2%) had a postoperative complication, and 486 (4.1%) had a 30-day readmission (Table 1). On multivariable logistic regression, those who were ≥80 years of age compared to <65 years of age had higher odds of same-day admission (OR: 2.31, 95% CI: 2.18-2.44, p < 0.0001), 30-day mortality (OR: 5.35, 95% CI: 3.21-8.92, p < 0.0001), 30-day composite postoperative complications (OR: 2.50, 95% CI: 2.17-2.88, p < 0.0001), and 30-day readmission (OR: 2.28, 95% CI: 2.05-2.53, p < 0.0001) (Table 2).

**Table 1:** Patient characteristics and postoperative outcomes for those undergoing the 10 most frequently performed general surgical procedures. COPD = chronic obstructive pulmonary disease.

	<65, n (%)	65-79, n (%)	≥80, n (%)	p-value
Surgical procedure				<0.0001
Laparoscopic cholecystectomy	68,874 (23.3)	13,363 (17.7)	2,218 (18.7)	
Laparoscopic appendectomy	57,267 (19.3)	4,595 (6.1)	536 (4.5)	
Partial mastectomy	21,978 (7.4)	15,181 (20.1)	2,636 (22.2)	
Laparoscopic repair inguinal hernia	25,791 (8.7)	11,114 (14.7)	1,810 (15.3)	
Inguinal hernia repair	23,013 (7.8)	12,066 (16.0)	3,118 (26.3)	
Umbilical hernia repair	26,779 (9.0)	4,803 (6.4)	475 (4.0)	
Knee arthroscopy	21,425 (7.2)	3,554 (4.7)	170 (1.4)	
Laparoscopic removal adnexal structure	21,053 (7.1)	1,379 (1.8)	151 (1.3)	
Shoulder arthroscopy	14,145 (4.8)	5,712 (7.6)	248 (2.1)	
Lumbar laminotomy	15,646 (5.3)	3,862 (5.1)	506 (4.3)	
Male sex	138,540 (46.8)	39,491 (52.2)	6,181 (52.1)	<0.0001
Morbid obesity (BMI ≥40kg/m^2^)	27,160 (9.2)	3,730 (4.9)	196 (1.7)	<0.0001
Diabetes mellitus				<0.0001
None	273,718 (92.5)	62,326 (82.4)	10,026 (84.5)	
Insulin-dependent	6,361 (2.1)	3383 (4.5)	458 (3.9)	
Non-insulin-dependent	15,892 (5.4)	9,920 (13.1)	1,384 (11.7)	
Active smoker	53,938 (18.2)	6,701 (8.9)	398 (3.4)	<0.0001
Dyspnea	5,327 (1.8)	3,948 (5.2)	996 (8.4)	<0.0001
Independent functional status	295,331 (99.8)	75,207 (99.4)	1,1581 (97.6)	<0.0001
COPD	3,571 (1.2)	3,372 (4.5)	677 (5.7)	<0.0001
Hypertension	66,748 (22.6)	45,018 (59.5)	8,608 (72.5)	<0.0001
Preoperative steroid use	4,579 (1.5)	2,136 (2.8)	333 (2.8)	<0.0001
Preoperative bleeding disorder	2,078 (0.7)	1,891 (2.5)	550 (4.6)	<0.0001
Hospital admission (length of stay >0 days)	61,936 (20.9)	10,318 (13.6)	2,408 (20.3)	<0.0001
Mortality	63 (0.0)	45 (0.1)	29 (0.2)	<0.0001
Complications	2,727 (0.9)	963 (1.3)	258 (2.2)	<0.0001
Readmissions	4,660 (1.6)	1,684 (2.2)	486 (4.1)	<0.0001

**Table 2:** Multivariable analysis of varying age groups with postoperative adverse events for all procedures.

Postoperative outcomes	OR (95% CI)	p-value
Hospital stay (>0 days)		
<65	Reference	
65-79	1.16 (1.13-1.20)	<0.0001
≥80	2.31 (2.18-2.44)	<0.0001
30-day mortality		
<65	Reference	
65-79	1.68 (1.10-2.56)	0.02
≥80	5.35 (3.21-8.92)	<0.0001
30-day composite complications		
<65	Reference	
65-79	1.51 (1.39-1.64)	<0.0001
≥80	2.50 (2.17-2.88)	<0.0001
30-day readmission		
<65	Reference	
65-79	1.33 (1.24-1.41)	<0.0001
≥80	2.28 (2.05-2.53)	<0.0001

We performed a separate multivariable regression analysis for each specific surgical procedure. Patients who were ≥80 years old had higher odds of same-day admission for laparoscopic cholecystectomy, partial mastectomy, laparoscopic inguinal hernia repair, inguinal hernia repair, umbilical hernia repair, laparoscopic removal of adnexal structures, and lumbar laminotomy, with the highest odds ratio being for the laparoscopic removal of adnexal structures (OR: 4.05, 95% CI: 2.80-5.86, p < 0.0001) (Table 3).

**Table 3:** Multivariable analysis of varying age groups with postoperative adverse events by surgical type.

Postoperative outcomes	OR (95% CI)	P-value
Laparoscopic cholecystectomy		
Hospital stay (>0 days)		
<65	Reference	
65-79	1.09 (1.03-1.14)	0.001
≥80	2.01 (1.83-2.21)	<0.0001
30-day mortality		
<65	Reference	
65-79	1.54 (0.78-3.07)	0.21
≥80	3.93 (1.63-9.50)	0.002
30-day complications		
<65	Reference	
65-79	1.73 (1.48-2.03)	<0.0001
≥80	2.43 (1.86-3.16)	<0.0001
30-day readmissions		
<65	Reference	
65-79	1.41 (1.26-1.57)	<0.0001
≥80	2.34 (1.95-2.8)	<0.0001
Laparoscopic appendectomy		
Hospital stay (>0 days)		
<65	Reference	
65-79	0.78 (0.73-0.84)	<0.0001
≥80	1.15 (0.95-1.39)	0.17
30-day mortality		
<65	Reference	
65-79	5.57 (0.83-37.3)	0.08
≥80	16.7 (1.3-218.8)	0.03
30-day complications		
<65	Reference	
65-79	1.07 (0.84-1.35)	0.59
≥80	1.50 (0.89-2.53)	0.13
30-day readmissions		
<65	Reference	
65-79	1.19 (0.98-1.43)	0.08
≥80	1.77 (1.19-2.63)	0.005
Partial mastectomy		
Hospital stay (>0 days)		
<65	Reference	
65-79	0.97 (0.88-1.07)	0.51
≥80	1.38 (1.17-1.63)	0.0001
30-day mortality		
<65	Reference	
65-79	0.50 (0.04-6.2)	0.59
≥80	5.12 (0.62-42.35)	0.13
30-day complications		
<65	Reference	
65-79	1.13 (0.88-1.44)	0.34
≥80	2.51 (1.76-3.57)	<0.0001
30-day readmissions		
<65	Reference	
65-79	0.92 (0.77-1.10)	0.37
≥80	1.36 (1.02-1.82)	0.04
Laparoscopic inguinal hernia repair		
Hospital stay (>0 days)		
<65	Reference	
65-79	1.77 (1.59-1.98)	<0.0001
≥80	4.00 (3.41-4.69)	<0.0001
30-day mortality		
<65	Reference	
65-79	1.88 (0.67-5.2)	0.23
≥80	4.85 (1.34-17.6)	0.02
30-day complications		
<65	Reference	
65-79	1.95 (1.48-2.56)	<0.0001
≥80	2.21 (1.40-3.51)	<0.0001
30-day readmissions		
<65	Reference	
65-79	1.62 (1.32-1.99)	<0.0001
≥80	2.75 (2.01-3.76)	<0.0001
Inguinal hernia repair		
Hospital stay (>0 days)		
<65	Reference	
65-79	1.44 (1.28-1.61)	<0.0001
≥80	3.44 (2.99-3.96)	<0.0001
30-day mortality		
<65	Reference	
65-79	1.23 (0.38-4.03)	0.73
≥80	6.18 (1.92-19.9)	0.002
30-day complications		
<65	Reference	
65-79	1.89 (1.40-2.56)	<0.0001
≥80	4.12 (2.87-5.91)	<0.0001
30-day readmissions		
<65	Reference	
65-79	1.40 (1.14-1.73)	0.002
≥80	3.17 (2.46-4.08)	<0.0001
Umbilical hernia repair		
Hospital stay (>0 days)		
<65	Reference	
65-79	1.46 (1.26-1.70)	<0.0001
≥80	3.05 (2.28-4.07)	<0.0001
30-day mortality		
<65	Reference	
65-79	5.33 (0.78-36)	0.09
≥80	30.7 (3.6-261.3)	0.002
30-day complications		
<65	Reference	
65-79	1.43 (1.00-2.05)	0.05
≥80	2.40 (1.17-4.91)	0.02
30-day readmissions		
<65	Reference	
65-79	1.34 (1.03-1.75)	0.03
≥80	2.72 (1.66-4.47)	<0.0001
Knee arthroscopy		
Hospital stay (>0 days)		
<65	Reference	
65-79	0.97 (0.67-1.39)	0.85
≥80	1.47 (0.46-4.71)	0.52
30-day mortality		
<65	Reference	
65-79	2.03 (0.36-11.56)	0.43
≥80	7.65 e-08 (0-Inf)	1.00
30-day complications		
<65	Reference	
65-79	1.09 (0.73-1.63)	0.68
≥80	1.94 (0.60-6.3)	0.27
30-day readmissions		
<65	Reference	
65-79	1.69 (1.11-2.57)	0.01
≥80	1.91 (4.53-8.02)	0.38
Laparoscopic removal adnexal structures		
Hospital stay (>0 days)		
<65	Reference	
65-79	1.98 (1.68-2.34)	<0.0001
≥80	4.05 (2.80-5.86)	<0.0001
30-day mortality		
<65	Reference	
65-79	0.98 (0.06-16.6)	1.00
≥80	1.18 e-08 (0-Inf)	1.00
30-day complications		
<65	Reference	
65-79	1.35 (0.88-2.09)	0.17
≥80	3.09 (1.38-6.94)	0.006
30-day readmissions		
<65	Reference	
65-79	1.48 (0.94-2.32)	0.09
≥80	3.91 (1.81-8.43)	0.0005
Shoulder arthroscopy		
Hospital stay (>0 days)		
<65	Reference	
65-79	1.21 (1.03-1.41)	0.02
≥80	1.61 (0.94-2.75)	0.08
30-day mortality		
<65	Reference	
65-79	0.90 (0.15-5.22)	0.90
≥80	5.62 e-08 (0-Inf)	1.00
30-day complications		
<65	Reference	
65-79	1.68 (1.19-2.38)	0.003
≥80	1.96 (0.61-6.31)	0.26
30-day readmissions		
<65	Reference	
65-79	1.66 (1.22-2.25)	0.001
≥80	2.49 (0.99-6.26)	0.052
Lumbar laminotomy		
Hospital stay (>0 days)		
<65	Reference	
65-79	1.61 (1.49-1.74)	<0.0001
≥80	2.4 (2.00-2.89)	<0.0001
30-day mortality		
<65	Reference	
65-79	4.62e+07 (0-Inf)	1.00
≥80	0.16 (0-Inf)	1.00
30-day complications		
<65	Reference	
65-79	1.98 (1.43-2.74)	<0.0001
≥80	1.87 (0.92-3.79)	0.08
30-day readmissions		
<65	Reference	
65-79	1.40 (1.12-1.75)	0.003
≥80	1.48 (0.91-2.4)	0.12

## Discussion

In our retrospective cohort analysis, we demonstrated an association between age and postoperative outcomes following outpatient surgeries requiring general anesthesia in the United States. We looked at the 10 most common ambulatory surgical procedures in ACS NSQIP and concluded that, when compared to a reference non-geriatric cohort, those who were ≥80 years of age had increased odds for same-day admission, mortality, composite complications, and hospital readmission. It has been unclear whether age should be used as an exclusion criterion for the safety of outpatient surgery performed at freestanding ambulatory centers. This study suggests that age should be considered in determining case appropriateness at outpatient surgery centers.

In the United Kingdom, around 8% of the population is above 75 years of age, with surgery in this patient population accounting for nearly 23% of all surgeries performed in the United Kingdom [6]. Certain studies in the United Kingdom have explored the difference in 30-day and 90-day mortality in patients aged ≥90 undergoing elective versus emergency surgical procedures, and have pointed to a more acceptable mortality rate for elective procedures. However, further studies with additional age ranges and postoperative outcomes can be helpful to truly understand the perioperative risk for elderly patients [7].

The indications for ambulatory surgery have rapidly evolved in light of the demands of modern medicine, healthcare services, and healthcare costs [8]. Increasingly and not surprisingly, outpatient surgery is being offered to higher risk and elderly patients. Several advantages of ambulatory surgery have been previously demonstrated including early mobilization, quicker return to the preoperative physiologic state, lower incidence of complications in addition to cost-effectiveness, and higher patient satisfaction [9]. Additionally, a study by Canet et al. suggested less cognitive dysfunction in the elderly undergoing minor ambulatory surgery within the first week postoperatively with avoidance of hospitalization when possible in older patients [10]. Given the advantages of ambulatory surgery, it is increasingly important to further define the patient population that would benefit the most from this setting.

There is no clear definition of age when a patient becomes an older person [11]. It is widely accepted that a person is defined as older or elderly when they are 65 years of age or greater. Given the increasing numbers in population of this group, it may be of benefit to further delineate age groups beyond greater than 65 compared to age groups less than 65 when assessing patients for ambulatory surgery.

In our study, age groups were categorized as <65 years old, between 65 and 79 years old, and ≥80 years old. We have identified that age ≥80 years old is an independent risk factor for hospital admission, 30-day mortality, 30-day readmission, and 30-day composite postoperative complications after ambulatory surgery. These results are consistent with prior literature. De Oliveira et al. demonstrated an association between age (<70 vs. ≥70) and hospital admission within 30 days after ambulatory surgery using the 2012 NSQIP dataset [12]. Older age was independently associated with a higher rate of hospital admission after ambulatory surgery and hospital admissions in general without post-surgical complications [12]. Older age has been shown to be an independent predictor of postsurgical morbidity, and hospital admission is more likely in individuals with morbidity [13]. Similarly, an administrative study by Fleisher et al. suggested that patients with advanced age (>85 years old) and history of prior inpatient admissions were at higher risk of hospitalization after ambulatory surgery, although the cause of inpatient hospitalization could not be determined [14].

Using the ACS NSQIP database, the overall mortality rate for those aged ≥80 years was 0.2%, and this age group was found to have a five-fold increase in risk for a mortality event. Age has been previously shown to be associated with a higher risk for cardiac and non-cardiac complications and in-hospital mortality in patients greater than 80 years of age. However, it should be noted that the rate of mortality does not exclusively preclude patients with advanced age from undergoing ambulatory surgery [15]. The combination of older age and comorbidities such as diabetes mellitus, cardiovascular disease, active smoking history, chronic obstructive pulmonary disease, hypertension, preoperative steroid use, and preoperative bleeding disorder place patients at increased risk for complications [16]. Additional factors such as functional status prior to surgery should be taken into account in assessing patients for ambulatory surgery.

Thirty-day postoperative complications were defined as the composite occurrence of superficial surgical site infection, wound disruption, pneumonia, reintubation, venous thromboembolism, urinary tract infection, stroke, or myocardial infarction. Cognitive dysfunction is common after anesthesia and in conjunction with decreased health literacy in the elderly may also contribute to hospital admissions and complications [17,18].

The results of the present study should be interpreted in the context of its limitations. This study implemented a retrospective design, which has certain inherent limitations such as the inability to account for all unknown confounders and non-uniformity in data collection. Given that the ACS NSQIP is a national database gathering information from numerous institutions, the differences in quality of care between facilities cannot be controlled for. The ACS NSQIP dataset does not identify whether the surgeries were performed at an ambulatory surgery center, although the procedures were scheduled as outpatient. Additionally, the characteristics of the hospitalizations within 30 days of procedures are not known. The specific outcomes related to a given procedure are also not known. However, the large sample size remains reassuring. Additionally, we were unable to collect more specific details regarding our patients’ comorbidities, such as recent myocardial infarction, symptomatic heart disease, and uncontrolled lung disease, which could have been added to the multivariate analysis to control for additional variables. Nonetheless, we included several comorbidities into our analysis, with all odds ratios continuing to remain statistically significant.

## Conclusions

Increasing age, particularly greater than 80 years or older compared to the cohort less than 65 years of age, is associated with at least two-fold increased odds of same-day hospital admissions, complications, and 30-day readmission after ambulatory surgery. Higher mortality was also noted in the age group greater than 80 years compared to the age group less than 65 years. Although we show that older patients are more likely to have cardiovascular (i.e., hypertension) and pulmonary comorbidities (i.e., dyspnea and chronic obstructive pulmonary disease), with impaired functional status, the association of older age with outcomes (i.e., hospital admission, complications, mortality, and readmission) remains after controlling for these variables in the multivariable model. This highlights the complexity of the physiology associated with aging. While age alone should not exclude elderly patients from ambulatory surgery, a thorough preoperative evaluation with intraoperative and postoperative planning may help to decrease poor outcomes in this population. Moreover, as the number and complexities of surgeries continue to increase in the ambulatory care setting, it is important for clinicians to be able to further delineate patients at higher risk of adverse events to improve the safety and care of older patients.
